# Editorial: Comorbidity in bipolar disorder, volume II

**DOI:** 10.3389/fpsyt.2023.1115357

**Published:** 2023-02-28

**Authors:** Domenico De Berardis, Michele Fornaro, Claudia Carmassi

**Affiliations:** ^1^Department of Psychiatry, Azienda Sanitaria Locale 4, Teramo, Italy; ^2^School of Nursing, University of L'Aquila, L'Aquila, Italy; ^3^International Centre for Education and Research in Neuropsychiatry, Samara State Medical University, Samara, Russia; ^4^School of Medicine and Surgery, University of Naples Federico II, Naples, Italy; ^5^Department of Clinical and Experimental Medicine, University of Pisa, Pisa, Italy

**Keywords:** bipolar (affective/mood) disorders, comorbidity, anxiety, ADHD, substance use disorder, posttraumatic stress disorder (PTSD), dissociative symptoms, overweight/obese

Bipolar disorder (BD), with a lifetime prevalence of about 2 per cent in the general population and a recurrent course tending toward chronicity, represents one of the most severe, frequent and costly psychiatric disorders, characterized by significant rates of disability, a high incidence of suicidality and multiple medical and psychiatric comorbidities (1). In addition, literature reviews estimate that at least 50 per cent of BD patients meet the criteria for other mental or organic disorders, with obvious repercussions on diagnostic framing, treatment and healthcare costs (2).

The presence of comorbidities, a concept that originated in general medicine in the 1970s but which finds its fullest expression in psychiatry, becomes fundamental for any in-depth study of the etiopathogenic hypotheses, the prognostic judgement and, above all, for the relevant therapeutic strategies (3).

Alarming data on the co-presence of other disorders in bipolar patients come from both large community epidemiological studies and those conducted on clinical samples. For example, the National Epidemiologic Survey on Alcohol and Related Conditions (NESARC), conducted in 2001–2002, confirmed that substance use and psychiatric disorders continue to be highly comorbid, and, in particular, bipolar disorder was steadily associated with panic disorder, agoraphobia, posttraumatic stress disorder, and borderline, schizotypal and antisocial personality disorders (4). The National Comorbidity Survey Study (NCS) and various clinical studies also reported high rates of comorbidity in BD patients (5–7). The works carried out by the Stanley Foundation Bipolar Treatment Outcome Network on about 300 patients with BD-I and BD-II showed that 65% of these subjects had met the diagnostic criteria for at least one other Axis I disorder at some point in their lives, with an earlier onset of affective symptoms and a worse prognosis (8, 9). On the other hand, Strakowski et al. (10), in a famous study of inpatients suffering from DB, showed psychiatric comorbidity of about 40 per cent and general medical comorbidity of 20 per cent, with a higher frequency in the female sex.

The psychiatric disorders most commonly associated with BD are anxiety disorders, eating disorders, attention deficit hyperactivity disorder and substance (SUD) and alcohol abuse (2). It remains to be clarified whether substance abuse is a cause or a consequence of BD. Still, the association between the two pathologies leads to increased affective mood swings, prolonged intervallic phases, a higher prevalence of physical disorders and suicide attempts, and worse adherence to treatment (11).

Concerning the main theme of the Research Topic, Aguglia et al. conducted a cross-sectional study involving 556 patients with a primary diagnosis of BD (376 without SUD, 101 with SUD, and 79 with Polysubstance Use Disorder [polySUD]). They found that younger age, male gender, early age at onset, psychotic and residual symptoms, positive family history of psychiatric disorders, and use of benzodiazepines were significantly associated with polySUD in patients with BD. Interestingly, patients with BD and polySUD were more likely to take four or more medications, particularly benzodiazepines and other drugs. The Authors pointed out that particular attention on this specific subtype of patients with BD may help implement personalized pharmacological and psychosocial therapies integrating the different professional roles.

Following the line of substance abuse and intoxication, Swoboda et al. aimed to compare intoxications due to a suicide attempt with an antidepressant (AD) and antipsychotic (AP) agents, or both with those of other medications and alcohol to illustrate the toxicity potential of these substances in a general population treated for intoxication. They conducted a retrospective and naturalistic one-year registry study that included 105 patients treated for oral intoxication at the University Department of Emergency Medicine in Vienna, Austria. AD/AP intoxications were present in 26 patients, while in the control group (*n* = 79), non-AD/AP drugs (*n* = 54) and exclusively alcohol (*n* = 25) were the toxic agents. In addition, they found that patients with AD/AP intoxication were significantly more often transferred to the psychiatric department, while discharge to home was more likely in the control group. Luckily, study results suggested that the risk of a potentially life-threatening outcome in intoxication with AD/AP wasn't substantially higher than in other readily available toxic agents, in line with the advantageous risk/benefit ratio of newer ADs and APs.

Two papers on the Research Topic addressed the problem of comorbidity between ADHD and BD. In a perspective paper, Comparelli et al. focused on specific clinical and developmental dimensions to recognize and/or differentiate the pattern of ADHD across the course of BD from a nosological perspective. They concluded that treating concurrent ADHD and BD remains an unresolved challenge regardless of the phase of illness. From a developmental perspective, such treatment might require a staged approach. By staging the introduction of treatments, it's possible to reduce the risk of overmedicating patients, better assessing the effect of each treatment. On the other hand, in the case of real comorbidity, a hierarchical approach to treatment should be followed: BD should be treated first. In contrast, ADHD should be treated by combining ADHD medications and mood stabilizers after mood stabilization. Besides, proper mood stabilizing therapy can reduce the chance of positive mood episodes that might arise if psychiatrists only use ADHD-specific medications. That is why a hierarchical approach should be followed. In another interesting study, Nunez et al. evaluated demographic, clinical, treatment, and genetic differences between BD with and without ADHD comorbidity, extending this comparison to consider the onset of attention deficits. Among patients with BD (*N* = 2,198) enrolled in the Mayo Clinic Bipolar Biobank, the researchers identified those with ADHD diagnosed in childhood (BD + cADHD; *N* = 350), those with adult-onset attention deficit symptoms (BD + aAD; *N* = 254), and those without ADHD (*N* = 1,594). A subset of the clinical sample had genotype data available. They found that attention deficits are more prevalent in men and associated with lower employment rates. In line with previous studies, they found a higher prevalence of ADHD in the offspring of BD patients. In addition, BD+ ADHD patients showed significantly higher rates of family history of affective disorders and a higher prevalence of substance use disorders. Specifically, it was observed increased rates of alcohol use disorder and stimulant use. Besides a higher prevalence of anxiety and depression disorders in patients with attentional deficits and, in terms of treatment response to mood stabilizers, the BD + cADHD group had a significantly poorer response to lithium and lamotrigine. Study results showed that BD + cADHD was associated with more significant comorbidities and reduced response to mood-stabilizing treatments. The higher ADHD polygenic risk scores (PRSs) for the BD + cADHD group may reflect a more powerful influence of genetic factors on the early presentation of ADHD symptoms.

Studies have found that traumatic events that occurred during childhood, adolescence and adulthood are associated with an increased risk of developing BD, with a significant likelihood of suicide and psychotic evolution (12–14). As dissociative disorders are an influential group of trauma-related disorders, the co-occurrence of dissociative disorders (DD) and symptoms (DS) in bipolar disorder has been relatively understudied. Still, there is some evidence that this comorbidity may have significant mechanistic and clinical implications. Rajkumar wrote an interesting scoping review on the frequency and correlates of DS and DD in BD. He pointed out that a significant minority of patients (10–20%) with bipolar disorder might experience important DS, even during the euthymic phase. The overall severity of DS was higher in BD than in healthy controls and major depression but lower in BD compared to “trauma spectrum disorders” such as DD, complex PTSD and borderline personality disorder. The presence of DS might be associated with psychotic symptoms, suicide attempts, and a poorer response to treatment. DS also appeared to be related to the severity of childhood trauma in patients with BD. Thus, assessing DD and DS in a patient affected by BD would be helpful in everyday clinical practice to adequately address the treatment.

On the other hand, Hogg et al. started from the assumption that post-traumatic stress disorder (PTSD) is an established comorbidity in BD. They conducted a multi-center study comprising 79 adult participants with BD with a history of psychological trauma and reported baseline data from a trial registered in Clinical Trials (https://clinicaltrials.gov; ref: NCT02634372). Study findings provided further evidence of the lack of difference in how trauma symptoms were presented across BD subtypes and provided essential data regarding the high levels of trauma symptoms in BD subjects, even when criteria for a PTSD diagnosis weren't met. However, the evidence showed that there were few differences in clinical BD severity between the subjects with full PTSD and subsyndromal PTSD, although they found a possible tendency for appositive correlation between full PTSD and psychotic symptoms, as well as between sexual abuse and rapid cycling, which can be clinically helpful in the identification and treatment of both. In conclusion, the study findings highlighted the proper investigation to understand the impact of comorbidity with a history of psychological trauma in BD patients, including subsyndromal PTSD symptoms and underlined the importance of screening for psychological trauma in the BD population.

Recently, some studies have suggested a higher risk of developing metabolic syndrome in BD than in the general population, increasing the risk of cardiovascular morbidity (15, 16). Furthermore, BD patients are at high risk of being overweight and obese, suffer more frequently from type II diabetes mellitus, and have higher cardiovascular mortality rates than the general population (17). Yi et al. conducted a retrospective, cross-sectional study to investigate the prevalence and associated factors of obesity and overweight in a sample of 1,169 inpatients with BD in China. They found that the prevalence rates of obesity and overweight were 21.0% and 32.2%, respectively, and the duration of BD was significantly associated with obesity. Besides, in a binary logistic regression analysis, the duration of BD and the levels of uric acid, ALT, triglycerides, and LDL cholesterol were identified as predictors for obesity. In contrast, male sex and uric acid level were associated with a higher frequency of overweight. The results of the present study show a need to implement early screening, prevention and interventions for obesity and overweight in patients with BD.

Finally, from a mixed psychopathological and translational perspective, gastrointestinal (GI) symptoms are widespread in BD patients but relatively understudied. Guo et al. recruited 59 BD patients that were divided into two groups. Each group was assessed with the 24-item Hamilton Depression Rating Scale (HAMD-24) according to the presence or absence of GI symptoms and compared with healthy controls. Differential metabolites were identified and further analyzed using Metabo Analyst 3.0 to identify associated metabolic pathways. The results showed that BD patients with GI symptoms experience more severe symptoms than the metabolic pathways related to GI symptoms, which may be risk factors for gastrointestinal symptoms in BD patients. Moreover, researchers found that the total HAMD-24 scores in the GI symptoms group were more significant than that of the non-GI symptoms group, consistent with past research findings. Based on metabolomic analysis results, it was also found that the common disturbances metabolic pathways of both groups of patients jointly exhibited disorders of ketone body metabolism: ketone body metabolism might be involved in the inflammation, and oxidative stress may be one of the pathogeneses of BD. Besides, the unique disturbances in metabolic pathways of BD patients with GI symptoms were fatty acid biosynthesis and tyrosine metabolism. One can argue that the abnormalities of these two metabolic pathways may be related to the disturbance of the gut microbiome, and the gut microbiome has been implicated in multiple human chronic GI disorders.

In conclusion, this Research Topic has shed light on the problem of comorbidity and BD, and researchers have profound a remarkable effort to address this topic. We would thank all of them for this and hope that the new forthcoming issue on Frontiers in Psychiatry entitled “Comorbidity in Bipolar Disorder and Schizophrenia Volume III” will further contribute to the understanding of comorbidity issues in severe psychiatric disorder, this time also in schizophrenia.

Finally, all the Guest Editors wish to dedicate the current Research Topic to Professor Gianna Sepede, MD PhD, a gifted psychiatrist whose kind manners and bright scientific and clinical skills inspired many of us as colleagues, friends, and trainees before her premature departure.

## Author contributions

All Authors have contributed to the present Editorial with equal efforts.
